# *MtTCP18* Regulates Plant Structure in *Medicago truncatula*

**DOI:** 10.3390/plants13071012

**Published:** 2024-04-02

**Authors:** Xiaoyue Su, Junzan Zheng, Xiaoxuan Diao, Zhongyi Yang, Deyue Yu, Fang Huang

**Affiliations:** Key Laboratory of Biology and Genetics Improvement of Soybean, Ministry of Agriculture, Zhongshan Biological Breeding Laboratory (ZSBBL), National Innovation Platform for Soybean Breeding and Industry-Education Integration, State Key Laboratory of Crop Genetics & Germplasm Enhancement and Utilization, Jiangsu Collaborative Innovation Center for Modern Crop Production, Nanjing Agricultural University, Nanjing 210095, China; 2020201048@stu.njau.edu.cn (X.S.); 2021101084@stu.njau.edu.cn (J.Z.); 2023801204@stu.njau.edu.cn (X.D.); t2023077@njau.edu.cn (Z.Y.); dyyu@njau.edu.cn (D.Y.)

**Keywords:** TCP transcription factors, plant structure, Tnt1 mutant, auxin, *Medicago truncatula*

## Abstract

Plant structure has a large influence on crop yield formation, with branching and plant height being the important factors that make it up. We identified a gene, *MtTCP18*, encoding a TEOSINTE BRANCHED1/CYCLOIDEA/PROLIFERATING CELL FACTOR (TCP) transcription factor highly conserved with *Arabidopsis* gene *BRC1* (BRANCHED1) in *Medicago truncatula*. Sequence analysis revealed that *MtTCP18* included a conserved basic helix–loop–helix (BHLH) motif and R domain. Expression analysis showed that *MtTCP18* was expressed in all organs examined, with relatively higher expression in pods and axillary buds. Subcellular localization analysis showed that MtTCP18 was localized in the nucleus and exhibited transcriptional activation activity. These results supported its role as a transcription factor. Meanwhile, we identified a homozygous mutant line (NF14875) with a mutation caused by Tnt1 insertion into *MtTCP18*. Mutant analysis showed that the mutation of *MtTCP18* altered plant structure, with increased plant height and branch number. Moreover, we found that the expression of auxin early response genes was modulated in the mutant. Therefore, *MtTCP18* may be a promising candidate gene for breeders to optimize plant structure for crop improvement.

## 1. Introduction

Plant structure is an important trait for crop growth and development, which is affected by several genes that control morphological traits such as plant height, number of nodes, internode length, and number of branches [1]. In recent years, genes that regulate plant structure have been frequently reported. In rice (*Oryza sativa*), the mutation of *OsSPL14* led to the “ideal” plant architecture with increased lodging resistance, reduced tiller number, and enhanced grain yield [2]. In soybean (*Glycine max*), *Dt2* mutant lines exhibited significantly increased branches and multiple yield-related trait changes, resulting in significantly increased yield [3]. Lack of *Rht-B1* (Reduced height-B1b) and *ZnF-B* (encoding ring-type E3 ligases) due to natural deletion formed semi-dwarf plants with more compact plant structure, which significantly increased seed yield in wheat (*Triticum aestivum*) [4].

Legumes are a large group of plants that are widely valued not only for their nutritional properties but also for their important role in food security and sustainable agriculture [5]. Model legumes are required for the study of developmental processes that cannot be studied in other model plants, such as the genetic control of inflorescence and flower development, compound leaf development, and symbiotic nitrogen fixation [5]. *Medicago truncatula*, as a model legume, is not only suitable for the study of the developmental processes but also has some advantages, including small diploid genome, autogamous nature, short generations, high transformation efficiency, etc. [5,6]. A library of mutants was created by researchers for *Medicago truncatula* using the Tnt1 retrotransposon of tobacco (*Nicotiana tabacum*) with R108 as the receptor background [7]. Many studies have utilized this mutant library to screen mutants by forward and reverse genetic methods and identified many functional genes involved in several physiological processes such as compound leaf development, flower development, and plant structure in *Medicago truncatula* [8,9,10].

TCP transcription factors have a TCP domain that has conserved function in the regulation of plant growth and development. In the gramineous plants, *ZmTB1* (TEOSINTE BRANCHED 1) in maize (*Zea mays*) acted as a repressor in branch outgrowth, with *OsTB1* and *TaTB1* functioning similarly [11,12,13]. In dicotyledons, *GmBRC1* was identified as controlling soybean branching by a genome-wide association study combined with linkage analysis [14]. In *Arabidopsis thaliana*, *AtBRC1* was also identified as a bud outgrowth repressor [15]. In addition, it was shown that TCP transcription factors were mainly involved in plant growth and development through the phytohormone signaling pathway. Overexpression of *SlTCP26* inhibited the expression of auxin-related genes to promote lateral branch development in tomato [16]. *CsBRC1* directly inhibited the function of *CsPIN3* (PIN-FORMED3), which led to the accumulation of auxin in cucumber axillary buds and inhibited the growth of lateral buds [17].

Indole-3-acetic acid (IAA) has profound effects on plant growth and development [18]. Since auxin works in a concentration-dependent manner and auxin gradients act as positional signals, plants have developed a complex system of auxin carriers to regulate auxin distribution [19]. The induction of *auxin*/*indole acetic acid* (*AUX*/*IAA*) genes is one of the hallmarks of the auxin response [20]. Some mutants of the *AUX*/*IAA* genes have been identified to be involved in the regulation of apical dominance in *Arabidopsis thaliana*, such as *iaa3*, *iaa7*, *iaa17*, and *iaa28* [21,22,23,24]. Some of these mutants have increased sensitivity to auxin, while others have decreased sensitivity. Unlike *Aux*/*IAA* proteins, *ARFs* (auxin factor response factors) contain a DNA-binding domain and bind to auxin response elements in the promoters of *Aux*/*IAA* genes and other auxin response genes [24,25,26]. In *Arabidopsis thaliana*, the phenotypes of the *arf7arf19* double mutant and the *iaa14*/*slr* mutant are very similar in lateral root development, and *ARF7* and *ARF19* show the same expression pattern as *IAA14* [27,28]. Therefore, *IAA14* may be a molecular chaperone of *ARF7* and *ARF19*, inhibiting the activity of these by forming a heterodimer in plants [28].

In this study, *MtTCP18*, a gene homologous to *Arabidopsis BRC1*, was identified in *Medicago truncatula* by the homologous cloning method. Phenotypic investigation of mutants clarified that *MtTCP18* altered plant structure. The relationship between *MtTCP18* and the auxin signaling pathway was investigated in *Medicago truncatula*.

## 2. Results

### 2.1. Identification of the TB1/BRC1 Ortholog in Medicago truncatula

To identify the putative orthologs of *AtBRC1* in *Medicago truncatula*, the full-length protein sequence of *AtBRC1* was used as a query in BLAST searches against the protein sequence database of the *Medicago truncatula* in Phytozome (https://phytozome-next.jgi.doe.gov/, accessed on 25 November 2021). Based on the homology analysis, *Medtr4g111935* (GenBank accession number: LOC25493935) displayed close relationships with *AtBRC1*/*TCP18* and was, therefore, named *MtTCP18*.

**Figure 1:**
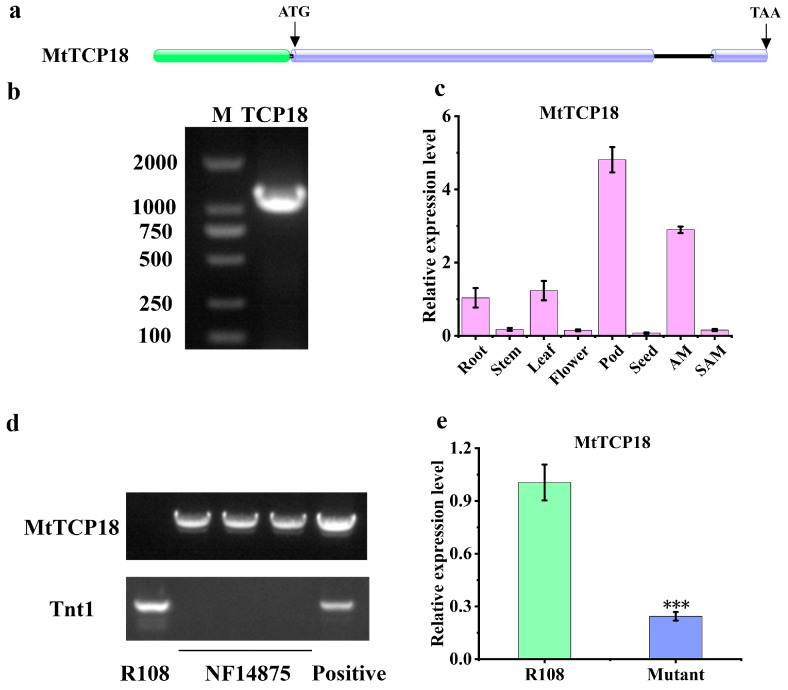
Gene structure, expression, and mutant characterization of *MtTCP18*. (**a**) The gene structure of *MtTCP18*. Green represents the 5’UTR, blue represents exons, black lines represent intron, and the black arrow indicates the location of ‘ATG’ and ‘TAA’. (**b**) Full-length cDNA amplicon from *MtTCP18*. The 2K marker is on the left, and the *MtTCP18* is on the right. (**c**) The expression of *MtTCP18* in various organs. (**d**) NF14875 mutant as analyzed by PCR and qRT-PCR. *MtTCP18*: specific primers spanning the insertion site; Tnt1: specific primers for Tnt1. R108 is the wild type and serves as a PCR negative control. (**e**) The expression of *MtTCP18* in WT and mutant. The relative expression levels were normalized to *Actin*. There were three biological replicates in qPCR. One-way ANOVA was used for statistical analysis. ***, *p* < 0.001; ns, not significant.

The sequence of *MtTCP18* was 1536 bp in length, including two exons and one intron (Figure 1a). The 1167 bp full-length cDNA of *MtTCP18* was amplified by RT-PCR from leaves (Figure 1b). To explore the expression pattern, we analyzed *MtTCP18* expression in roots, stems, leaves, flowers, pods, seeds, axillary buds (AM), and shoot apical meristems (SAM) by RT-PCR. The results showed that *MtTCP18* was expressed in all organs examined, with higher expression in pods and axillary buds (Figure 1c), which was similar to *OsTB1* and *TaTB1* with expression in axillary buds [12,29]. The expression pattern of *MtTCP18* suggests that it may play a role in axillary bud development in *Medicago truncatula*.

### 2.2. MtTCP18 Contains the Conserved Domains

*TB1*/*BRC1* is a TCP transcription factor that is a key hub for axillary bud inhibition in different plants [10]. The *Arabidopsis* gene, *BRC1*, encodes a TCP transcription factor, which is closely related to *TB1* in maize [11]. *BRC1* represents a key point at which signals controlling branches are integrated within axillary buds [15]. In rice, RNAi-mediated knockdown of *OsTB1* resulted in the reduction of a number of tillers and panicles [30]. Overexpressing *OsTB1* exhibited reduced lateral branching [12].

**Figure 2:**
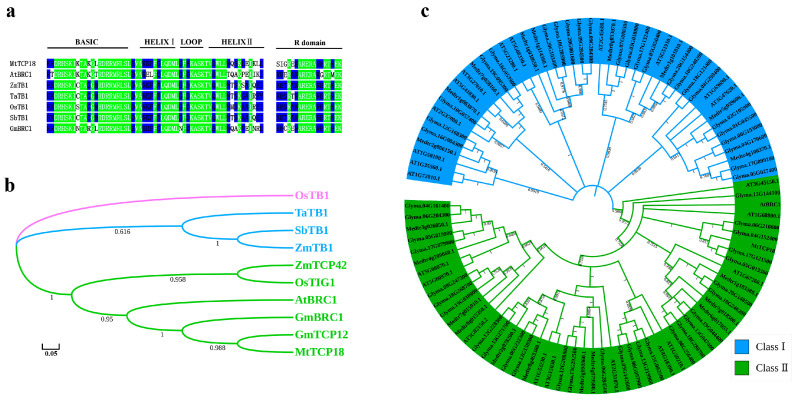
Sequence alignment and phylogenic analysis of MtTCP18. (**a**) Amino acid sequence alignments of TCP members in different plants. Green is the consistent background color; Blue is conservative background color. (**b**) The phylogenetic tree was generated using ClustalW via sequence alignment of *MtTCP18* and other TCP members in different plants. *GmBRC1* (Glyma.06G210600) and *GmTCP12* (Glyma.17G121500) are TCP members of soybean. *OsTB1* (XP015630237) and *OsTIG1* (LOC_Os08g33530) are TCP members of rice. *ZmTB1* (Zm00001d033673) and *ZmTCP42* (Zm00001d022256) are TCP members of maize. *AtBRC1* in *Arabidopsis*, *TaTB1* (XP044372296) in wheat, and *SbTB1* (XP002466597) in *Sorghum bicolor* are TCP members. (**c**) Phylogenic analysis of TCP members in *Medicago truncatula*, *Arabidopsis*, and soybean. The phylogenetic tree was generated by MEGA_X_10.1.7 after sequence alignment of MtTCP18 and other TCPs using ClustalW.

In this study, *MtTCP18*, *AtBRC1*, *ZmTB1*, and other reported genes in different plants were analyzed by amino acid sequence comparison and evolutionary analysis. We found that these genes encode putative transcription factors carrying a bHLH type of DNA-binding motif, named the TCP domain (Figure 2a,b). There are 21 TCP family members in *Medicago truncatula*. We performed evolutionary analysis of TCP family members in *Arabidopsis thaliana*, *Medicago truncatula*, and soybean. The results showed that the TCP families in dicotyledons were divided into two classes, Class Ⅱ of which included the TB1-like genes (Figure 2c).

### 2.3. MtTCP18 Encodes a Nuclear Protein Functioning as Transcriptional Activator

**Figure 3:**
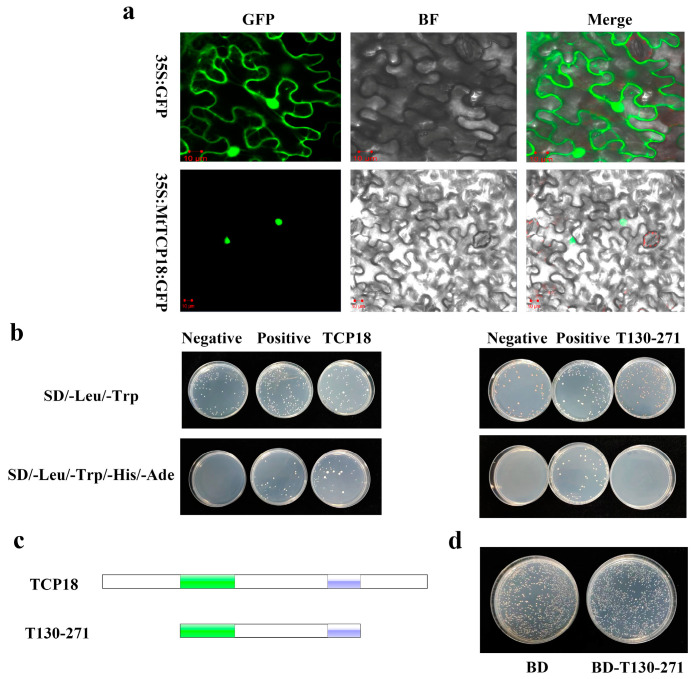
Molecular identification of MtTCP18 as a transcription factor. (**a**) Subcellular localization of MtTCP18. GFP: green fluorescent protein; BF: bright field. Scale bars: 10 µm. (**b**) Transcriptional activation activity of MtTCP18 in yeast. TCP18 represented the full-length protein of *MtTCP18*, while T130-271 represented the truncated protein of *MtTCP18*, both of which were validated on BD vectors. (**c**) Construction of BD vector for *MtTCP18*. Green represents the conserved bHLH domain, while blue represents the R domain. (**d**) Yeast toxicity experiment of *MtTCP18*. Transform of BD empty and T130-271 into yeast and culture for 2–3 days on SD/-Trp medium.

Many biological processes are fine-tuned at the transcriptional level, which is primarily accomplished by regulating the activity of transcription factors [31]. GFP-tagged *MtTCP18* coding sequences (CDS) driven by the CaMV 35S promoter (35Spro:MtTCP18-GFP) were generated and transiently expressed in tobacco leaves. MtTCP18-GFP fusion protein was localized in the nucleus, which supports its role as a transcription factor (Figure 3a).

Moreover, we validated the transcriptional activation activity of MtTCP18 by the yeast hybrid assay. The full-length protein of MtTCP18 exhibited transcription activation activity in the yeast cells, while the truncated protein with the amino acid sequence from 130-271 (named T130-271), that retained only the TCP domain and R domain, did not (Figure 3b,c). The truncated protein was subjected to toxicity assays, and we found that it had no effect on yeast growth (Figure 3d).

### 2.4. The Mutation of MtTCP18 Increases Plant Height and Branch Number in Medicago truncatula

**Figure 4:**
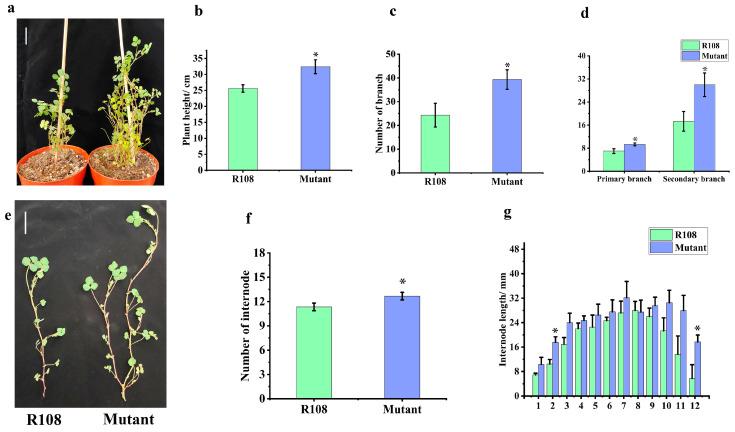
Characterization of the NF14875 mutant. (**a**,**e**) The plant structure of R108 (WT) and NF14875 mutant at the beginning of the flowering period. (**a**) scale bar: 5 cm. (**e**) scale bar: 3 mm. Comparison of plant height (**b**), the number of branches (**c**,**d**), number of internodes (**f**), and length of internodes (**g**) for WT and mutant. Data are measured with three biological replicates. One-way ANOVA was used for statistical analysis. *, *p* < 0.05.

Tnt1 was one of the few well-characterized long terminal repeat retrotransposons in plants [32]. Tnt1 was relatively active during tissue culture but was stable during seed-to-seed propagation, so it was chosen to initiate near-saturation mutagenesis in *Medicago truncatula* [7,33]. To determine the possible role of *MtTCP18* in affecting plant structure in *Medicago truncatula*, we identified a mutant line NF14875 with Tnt1 retrotransposon insertion (Figure 1d). In the homozygous mutant line, the expression of *MtTCP18* was significantly reduced (Figure 1d,e). We investigated the height and branch number of WT and mutant at the beginning of flowering period. The mutant showed a significant enhancement in plant height, due to the increase both in number and length of internodes (Figure 4a,b,e–g). In addition, the primary and secondary branches increased in the mutant, leading to an improvement in the total number of branches (Figure 4a,c,d). Because *MtTCP18* was highly expressed in the pods, we measured the length of the diameter and thickness in the pod. Our data showed that diameter and thickness did not change significantly (Supplemental Figure S1).

### 2.5. NF14875 Showed Altered Auxin-Related Gene Expression

**Figure 5:**
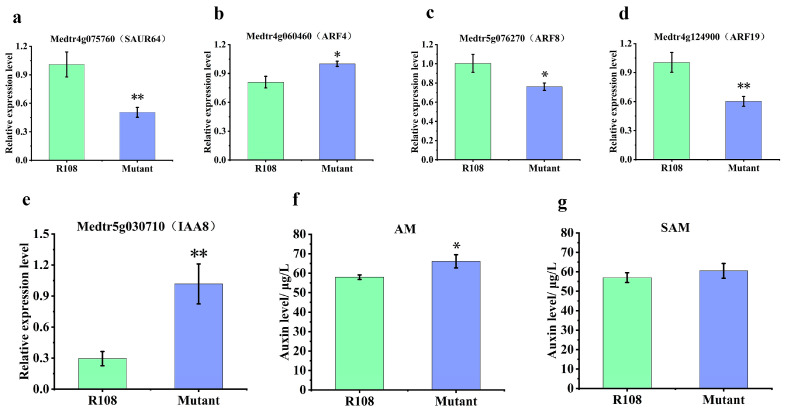
Expression of auxin-related genes and determination of endogenous auxin level in WT and NF14875 mutant. (**a**–**e**) Validation of the expression patterns of auxin-related genes by qRT-PCR. The relative expression level was normalized to *Actin*. (**f**,**g**) The IAA contents were detected by an enzyme-linked immunosorbent assay (ELISA) kit specific for IAA. Data were measured with three biological replicates. One-way ANOVA was used for statistical analysis. *, *p* < 0.05; **, *p* < 0.01. AM, axillary buds; SAM, shoot apical meristems.

SAM supports vertical growth in plants, while AM occurs in the axils of leaves and can produce lateral branches [34]. TCP transcription factors have been reported to regulate plant structure by influencing phytohormone signaling pathways, including auxin. In *Arabidopsis thaliana*, *AtTCP3* activated the expression of genes such as *IAA3* and *SAUR*, which negatively acted on the formation of shoot meristems [35]. Overexpression of *AtTCP15* reduced the auxin level by suppressing the expression of the auxin biosynthesis genes *YUCCA1* and *YUCCA4* [36]. In order to determine the relationship between *MtTCP18* and the auxin signaling pathway, we probed the expression of auxin- related genes in WT and NF14875. In *Arabidopsis*, the mutation *BOLITA* (*BOL*), an *AP2*/*ERF* transcription factor, resulted in the suppression of *SAUR64* expression in leaves [37]. *AS2* (ASYMMETRIC LEAVES2) is responsible for the development of flat, symmetric, and extended leaf laminae and their vein systems. *AS2* forms a complex with *AS1*, which is involved in epigenetic repression of the *ARF3*, *ARF4*, and class 1 *KNOX* homology cassette genes [38]. *ARF6* and *ARF8* activated the transcription of *DWARF4*, which encoded a key brassinosteroid (BR) biosynthetic enzyme in the regulation of leaf shape [39]. *ARF19* is a transcriptional activator that regulates auxin-mediated transcriptional and developmental responses in *Arabidopsis* [27,28]. *IAA8* negatively regulated lateral root formation but also promoted seed germination [40]. The results showed significant differences in the expression of early auxin response genes in NF14875, including *ARFs*, *AUX*/*IAA*, and *SAUR* (Figure 5a–e). Moreover, we found that the content of auxin in the shoot meristems and axillary buds increased in the NF14875 mutants, by measuring the IAA (Figure 5f,g).

## 3. Discussion

TCP family members have a strong influence on the growth patterns during plant development and are therefore key determinants of plant morphology. In recent years, some TCP transcription factors have been reported to be involved in biological processes such as leaf development, flower morphology, and bud formation. Overexpression of five miRNA-down-regulating TCP genes resulted in heterogeneous leaf traits and curvature in *Arabidopsis thaliana* [41]. The *tcp15* mutants had shorter petioles in *Arabidopsis* [42], while Kieffer et al. (2011) found that *tcp14tcp15* double mutants exhibited another slight leaf defect [43]. In eudicots, bilateral floral symmetry is regulated by the TCP protein family of *TB1*/*CYC* transcription factors [44,45]. Moreover, in *Gerbera hybrida*, the E class MADS-box transcription factor GRCD5 targets *GhCYC3*, thereby affecting stamen development [46]. *TB1*/*BRC1*-like genes are functionally more conserved in monocots such as maize and rice and in dicotyledons such as *Arabidopsis* and *Medicago truncatula*, both of which are involved in the regulation of lateral bud formation (Figure 4) [11,15]. In this study, although only one mutant line demonstrated that *MtTCP18* affected the lateral branch formation, its phenotypic traits were also expected based on the literature [11,12,13,14,15,17]. This is not a fully rigorous way to proceed but it can be acceptable for a phenotype well established in other species, which is confirmed for *Medicago truncatula*. However, whilst MtTCP18 acts as a transcription factor, the molecular mechanism is currently unknown. IPA1 (IDEAL PLANT ARCHITECTURE1) is a transcription factor that promotes the expression of *OsTB1* by directly binding to its promoter region [47]. *TIE1* (TCP interactor containing EAR motif protein1) did not only interact with *BRC1* in vivo and in vitro: it directly inhibited BRC1 activity to regulate stem meristems [48]. Three HD-ZIP transcription factor-encoding genes, *HB21* (HOMEOBOX), *HB40*, and *HB53*, were shown to be directly regulated by BRC1 in *Arabidopsis thaliana* [49]. Due to the highly conserved domains of the TB1/BRC1, we hypothesized a *Medicago* homologue of IPA1 targets binding to *MtTCP18*, which in turn binds to *HB21*, *HB40*, and *HB53* to regulate lateral branch growth.

Moreover, TCP family members are classified into two groups (Class Ⅰ and Class Ⅱ) based on the structure of their conserved DNA binding and dimerization domains [50,51]. In *Arabidopsis*, Class I is formed by 13 predicted proteins associated with the *PCF* rice factors, and Class II is formed by 11 predicted proteins associated with the *CYC* and *CIN* genes as well as *TB1* [15]. In *Medicago truncatula*, Class I showed conserved exon-intron organization but Class II showed variable numbers of introns [52]. In soybean, TCP family members were highly conserved, and the results for subclasses were consistent with those of *Arabidopsis thaliana* and *Medicago truncatula* (Figure 2c). Soybean is one of the major crops that provides the oil and proteins for humans and animals [53], as expressed in a Chinese saying, “you won’t need to take medicine for years if you eat 15 g of soybeans a day” [54]. However, its yield is obviously lower than other crops such as maize and rice. This is significant for identifying the important genes regulating yield-related traits in soybean [55]. Two homologous genes (*Glyma.05G013300* and *Glyma.17G121500*) in soybean are highly homologous to *MtTCP18* and may regulate the formation of plant height and lateral branch, affecting soybean yield.

TCP transcription factors affect plant growth and developmental processes by participating in the biosynthesis of plant hormones and through direct regulation of signaling pathways, including jasmonic acid (JA), gibberellin (GA), cytokinins (CK), abscisic acid (ABA), BR, and auxin. *BrTCP21* binds to the promoter region of the GA biosynthesis gene *BrGA20ox3* and is involved in the regulation of leaf senescence through activation of the GA biosynthesis pathway in Chinese flowering cabbage [56]. In *Fragaria vesca*, FvTCP9 promotes fruit ripening by regulating the biosynthesis of ABA signaling-related genes (*FaNCED1*, *FaPYR1*, *FaSnRK2*, and *FaABI5*) and anthocyanins [57]. *GrTCP11*, a homolog of *Arabidopsis* AtTCP11, may be an important transcription factor for cotton fiber development and affects in vivo JA levels by negatively regulating JA biosynthesis and response genes in diploid cotton (*Gossypium raimondii*) [58]. In *Arabidopsis*, TCP8 directly binds to and transcriptionally activates key BR gene promoters in plants, and *TCP8* activity and subcellular localization are dependent on BR [50]. It has been frequently reported that TCP family members participate in the auxin signaling pathway [16,35,36]. Meanwhile, GhTCP14 directly targets the promoters of *PIN2*, *IAA3*, and *AUX1*, thereby mediating auxin-mediated epidermal cell development in cotton (*Gossypium hirsutum*) [59]. In *Medicago truncatula*, *MtTCP18* regulates the expression of *ARFs*, SAURs, and *IAAs* genes, and it is hypothesized that MtTCP18 may directly target the regulation of some of these genes (Figure 5a–e). The auxin content of axillary bud and shoot apical meristems was increased, which was suggested to be responsible for the increase in plant height and lateral branches (Figure 5f,g). This suggests that MtTCP18 suppressed auxin synthesis to regulate plant structure by targeting the expression of auxin-related genes.

## 4. Materials and Methods

### 4.1. Plant Materials and Growth Conditions

The wild-type *Medicago truncatula* ecotype R108 and mutant were grown in a glasshouse at 24 °C:22 °C (day:night) with a photoperiod of 16 h:8 h (light:dark) in a mixture of soil and sand (1:1). We investigated plant height, number of internodes, internode length, and number of branches for WT and mutants at the beginning of the flowering period (64 days). Pods were harvested from single plant material, which were measured for diameter and thickness.

### 4.2. Identification of Tnt1 Mutants

One mutant line, NF14875, with insertion in *MtTCP18*, was identified by a BLAST search on the *Medicago truncatula* mutant library (https://medicago-mutant.dasnr.okstate.edu/, accessed on 25 November 2021) [7]. A primer pair spanning the Tnt1 insertion (NF14875-F/R) and Tnt1-specific primers (Tnt1-F/R) were used to verify the presence of Tnt1 insertion and its homozygous/heterozygous condition via PCR analysis. The primers are listed in Supporting Information Table S1.

### 4.3. Cloning of the MtTCP18 Gene

In this study, genomic DNA was extracted using a DNAsecure Plant Kit (TIANGEN, Beijing, China). The leaves of R108 were taken separately. The samples were immediately frozen in liquid nitrogen and stored at −80 °C in an ultra-low-temperature refrigerator for use. Total RNA was extracted with an RNA extraction kit (RNAsimple Total RNA Kit, CWBIO, Taizhou, China), and first-strand cDNA was obtained with a reverse transcriptase kit (DNAsecure Plant kit, TIANGEN, Beijing, China). The quality and concentration of DNA and RNA were assayed by gel electrophoresis and spectrophotometry. Based on the mRNA sequence of *Medtr4g111935* on Phytozome (https://phytozome-next.jgi.doe.gov/, accessed on 25 November 2021, *Medicago truncatula* Mt4.0v1), the cDNA sequence of *MtTCP18* was cloned by using specific primers. The procedure for cDNA cloning was as follows: initial denaturation for 3 min at 95 °C, followed by 35 cycles each consisting of denaturation for 15 s at 95 °C, annealing for 15 s at 58 °C, and extension for 45 s at 72 °C; the final cycle was extended for 5 min at 72 °C. The cDNA sequence of *MtTCP18* was ligated into an entry T vector using pClone007 Blunt Simple Vector Kit (TsingKe, Beijing, China, TSV-007BS); then, the recombinant T plasmid with *MtTCP18* cDNA was named as T-MtTCP18 and confirmed by sequencing.

### 4.4. Phylogenetic Analysis and Alignment of Protein Sequences

To conduct phylogenetic analysis, 24 *Arabidopsis thaliana* sequences, 21 *Medicago truncatula* sequences, and 53 soybean sequences annotated as TCPs from the websites named PlantTFDB (http://planttfdb.gao-lab.org/, accessed on 10 October 2022) were retrieved [60]. The TCP protein sequences of different plants were analyzed by ClustalW [61]. Phylogenetic trees of TCP family members in dicotyledonous plants were constructed with the neighbor-joining algorithm using the MEGA_X_10.1.7 program, with bootstrapping values set to 500 replicates [62]. These phylogenetic trees were visualized with the online tool iTOL (https://itol.embl.de, accessed on 12 December 2022) [63].

### 4.5. qRT-PCR Analysis

Total RNA was extracted with an RNA extraction kit (RNAsimple Total RNA Kit, CWBIO, Jiangsu), and first-strand cDNA was obtained with a reverse transcriptase kit (DNAsecure Plant kit, TIANGEN, Beijing, China). The roots, stems, leaves, flowers, pods, seeds, axillary buds (AM), and shoot apical meristems (SAM) of R108 and the leaves of mutants (68 days) were taken separately. The samples were immediately frozen in liquid nitrogen and stored at −80 °C in an ultra-low-temperature refrigerator for use. We examined the expression of the *MtTCP18* and auxin-related genes by utilizing specific primers. The mRNA level of the *Actin* was used as a quantitative control. The primers are listed in Supporting Information Table S1.

### 4.6. Subcellular Localization Analysis

The full-length CDS of *MtTCP18* (excluding stop codon) was amplified with two primers *MtTCP18*-SL-F/R (Table S1) and cloned into the pFGC5941 vector to generate a fusion construct with the coding sequence of green fluorescent protein (GFP) [62]. The resulting constructs (35S: GFP, as a control vector, and 35S: *MtTCP18*: GFP) were introduced into *Agrobacterium tumefaciens* strain EHA105. Tobacco leaves were grown for 20–30 days and infested with *Agrobacterium tumefaciens*. The infested tobaccos plants grew in darkness for 24 h, after which they grew in light for 1–2 days. Finally, the injected tobacco leaves were imaged using a confocal laser scanning microscope (LSM780; Zeiss, Jena, Germany).

### 4.7. Transcriptional Activity Assay

The transcriptional activation activity of MtTCP18 was assessed by utilizing a yeast two-hybrid system. The full-length and truncated sequences of *MtTCP18* were ligated into the BD vector (pGBKT7) using the homologous recombination system. The primers are listed in Supporting Information Table S1. BD-*MtTCP18*, truncated BD-*MtTCP18* (130–271), AD (pGADT7), and positive and negative control vectors were, respectively, introduced into yeast Y2H [62]. At a temperature of 30 °C, the transformed yeast cells were grown on two defective mediums (SD/-Trp/-Leu and SD/-Trp/-Leu/-His/-Ade) for 4–5 days; the purpose was to verify *MtTCP18* transcriptional activation activity. At a temperature of 30 °C, the transformed yeast cells were grown on the defective medium (SD/-Trp) for 4–5 days; the purpose was to verify whether *MtTCP18* affected yeast growth.

### 4.8. Auxin Content Determination

The samples of axillary buds and shoot meristems for WT and mutants were collected at the growing stage (83 days) in Nanjing. To measure the auxin content, 0.1 g samples were ground with liquid nitrogen and then transferred into a 2.0 mL tube containing phosphate buffer.

Auxin was extracted according to the protocol of Duan et al. (2023), and its content was measured using an enzyme-linked immunosorbent assay (ELISA) kit specific for IAA (Meimian Inc., Shanghai, China) [64]. The experiments were performed with 3 independent biological replicates.

## 5. Conclusions

In this study, we conducted a preliminary functional investigation of *MtTCP18* via homologous gene cloning, bioinformatics analysis, and some molecular experiments. The phenotypic characterization of the mutant revealed that MtTCP18 changed the plant structure by affecting the plant height and the number of branches. MtTCP18 affected the expression of auxin early response genes and participated in the auxin signaling pathway. Further research will focus on whether *MtTCP18* directly targets auxin-related genes and mediates auxin biosynthesis, thus refining the *MtTCP18* regulatory network. In addition, we will study the homologous genes of *MtTCP18* in soybean and create transgenic mutant lines via gene editing technology; this may be applied for breeding high-yield soybean cultivars.

## Data Availability

The data presented in this study are available in the Supplementary Materials.

## Supplementary Materials

The following supporting information can be downloaded at https://www.mdpi.com/article/10.3390/plants13071012/s1. Table S1: The sequence of primers. Table S2: TCP transcription factors in different plants. Table S3: TCP transcription factors in dicotyledonous plants. Supplementary Figure S1: Characterization of the mutant in pods.
