# Supplementary material for: MtTCP18 Regulates Plant Structure in Medicago truncatula
Source: Plants (Basel). 2024 Apr 2;13(7):1012. doi: 10.3390/plants13071012 (PMC11013128; doi:10.3390/plants13071012)
Supplement: Supplementary file 1 [file plants-13-01012-s001.zip › Supplemental figure 1.pdf]

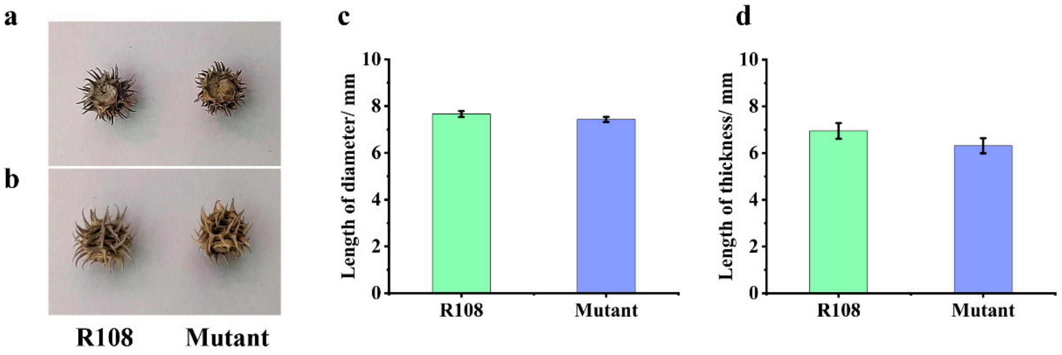

Supplemental figure 1. Characterization of the mutant in pods. (a)-(b) the pods of R108 (WT) and mutant at harvest. Comparison of length of diameter (c) and thickness (d) for WT and mutant. There are three biological replicates. One-way ANOVA were used for statistical analysis. *, $P < 0.05$; **, $P < 0.01$; ***, $P < 0.001$; ns, not significant.
